# *Escherichia coli* O157:H7 Curli Fimbriae Promotes Biofilm Formation, Epithelial Cell Invasion, and Persistence in Cattle

**DOI:** 10.3390/microorganisms8040580

**Published:** 2020-04-17

**Authors:** Haiqing Sheng, Yansong Xue, Wei Zhao, Carolyn J. Hovde, Scott A. Minnich

**Affiliations:** Bi-State School of Food Science, University of Idaho, Moscow, ID 83844-2312, USA; hqsheng@uidaho.edu (H.S.); yansong.xue@wsu.edu (Y.X.); weizhao_822@mail.tsinghua.edu.cn (W.Z.); sminnich@uidaho.edu (S.A.M.)

**Keywords:** curli, *E. coli* O157, biofilm, invasion, cattle

## Abstract

*Escherichia coli* O157:H7 (O157) is noninvasive and a weak biofilm producer; however, a subset of O157 are exceptions. O157 ATCC 43895 forms biofilms and invades epithelial cells. Tn*5* mutagenesis identified a mutation responsible for both phenotypes. The insertion mapped within the curli *csgB* fimbriae locus. Screening of O157 strains for biofilm formation and cell invasion identified a bovine and a clinical isolate with those characteristics. A single base pair A to T transversion, intergenic to the curli divergent operons *csgDEFG* and *csgBAC*, was present only in biofilm-producing and invasive strains. Using site-directed mutagenesis, this single base change was introduced into two curli-negative/noninvasive O157 strains and modified strains to form biofilms, produce curli, and gain invasive capability. Transmission electron microscopy (EM) and immuno-EM confirmed curli fibers. EM of bovine epithelial cells (MAC-T) co-cultured with curli-expressing O157 showed intracellular bacteria. The role of curli in O157 persistence in cattle was examined by challenging cattle with curli-positive and -negative O157 and comparing carriage. The duration of bovine colonization with the O157 curli-negative mutant was shorter than its curli-positive isogenic parent. These findings definitively demonstrate that a single base pair stably confers biofilm formation, epithelial cell invasion, and persistence in cattle.

## 1. Introduction

Enterohemorrhagic *Escherichia coli* (EHEC) cause human disease with symptoms ranging from self-limited watery diarrhea to life-threatening hemorrhagic colitis, and hemolytic uremic syndrome [1,2]. *E. coli* O157:H7 (O157) is the best studied EHEC serotype and is the predominant strain associated with disease outbreaks in North America, the United Kingdom, and Japan [3,4,5]. Cattle and other ruminants carry this pathogen with no apparent symptoms [6,7] and are the most common source for human infections [8,9]. O157 colonize at the bovine recto-anal junction (RAJ) and the bacteria persist in the feces of individual animals from a few days to several months [10,11]. Attachment to biological surfaces is a first critical step in colonization and is mediated by multiple bacterial factors. Surface-associated factors of O157 contributing to tissue adherence and persistence in the bovine host include O-antigen [12], fimbriae [13], adhesins such as intimin [14], and some autotransporters [15]. There is evidence that the duration of colonization and the bovine immune responses are strain/variant dependent [16,17].

Curli fimbriae, comprised of polymerized amyloid protein, are expressed on the surface of many members of the *Enterobacteriaceae* and other Gammaproteobacteria [18]. Curli binds amyloid-specific dyes, such as Congo red and certain host proteins, including fibronectin, laminin, and plasminogen [19,20]. During infections, curli complexes with extracellular matrix DNA. In a mouse model for lupus erythematosus autoimmunity, these curli-DNA complexes interact with Toll-like receptors (TLRs) 2 and 9 on dendritic and macrophage cells resulting in the production of autoantibodies [21]. In most non-O157 *E. coli*, curli is regulated by σ^s^ and synthesized at low temperature, in nutrient-deprivation, and/or in stationary phase, conditions that promote biofilm formation [22]. Curli synthesis requires the expression of genes from two divergently transcribed operons, designated *csgDEFG* and *csgBAC*. Genes with identified functions include the regulator CsgD, the type VIII secretion machinery components CsgE-G, the curli major subunit CsgA, and the curli nucleation protein CsgB [23,24]. The intergenic region between *csgDEFG* and *csgBAC* is large and contains many putative binding sites for regulatory factors. CsgD is essential for the transcription of the curli operons [19]. Curli promotes biofilm adhesion to abiotic surfaces as well as to mammalian cells [25,26,27,28]. Although both operons are present in all sequenced O157 strains [29,30,31], the majority of O157 (approximately 95%) are curli-negative. This is because the prophage carrying Shiga toxin type-1 inserts into *mlrA*, a positive regulator of *csgD* [32]. The few curli-positive O157 strains produce curli constitutively, including at 37 °C, and have acquired a suppressor mutation overriding the normal requirement for *mlrA* [33,34].

O157 is a weak biofilm producer and is considered an extracellular pathogen [35,36]. However, some strains do not meet this general characterization. In a previous study, we showed that O157 strain 43895, an outbreak isolate from hamburger, produces biofilms at 37 °C, invades epithelial cells, and persists longer in cattle than a biofilm-negative strain [16]. Curli expression has been found in certain O157 strains [34], but the underlying mechanism has not been fully explored.

We hypothesized that curli expression, biofilm formation, and cell invasion are genetically linked and cause persistence in cattle. To test this hypothesis, a library of strain 43895 Tn*5*-mutants was screened for loss of biofilm production and then the biofilm-negative mutants were screened for epithelial cell invasion and tested in cattle. Biofilm-negative mutants were due to either a loss of lipopolysaccharide (LPS) or curli synthesis. Only Tn*5*::*csgB*, a curli subunit gene insertion, affected both biofilm and cell invasion phenotypes. Moreover, this mutant did not persist in cattle as long as the parental wild type. Importantly, one of the biofilm-positive strains in our screen contained an identical A to T transversion present in the strain 43895 promoter of CsgD. Changing this single base pair (A to T transversion) in the *csgD* promoter converted curli negative strains to be stably curli positive, biofilm-forming, Congo red dye-binding, and invasive to epithelial cells.

## 2. Materials and Methods

### 2.1. Bacteria, Plasmids, Primers, and Growth Conditions

Bacteria, plasmids, and primers used in this study are shown in Table 1 and Table 2. Bacteria were grown in Luria-Bertani (LB) broth or agar at 37 °C, unless otherwise stated. These *E. coli* strains (wild type, mutants, complemented mutants, and others, did not differ in growth rates (data not shown)). When required, antibiotics kanamycin (KAN, 50 mg/mL), chloramphenicol (CHL, 30 mg/mL), or ampicillin (AMP, 100 mg/mL) were added to the media. Congo red dye-binding was monitored on Congo red indicator agar, according to Hammar et al. [24]:10 g Casamino acids, 1 g yeast extract, and 20 g agar (Difco, Detroit, MI, USA), 20 mg Congo red and 10 mg Coomassie brilliant blue G-250/L (Sigma-Aldrich, St. Louis, MO, USA). The lambda red recombinase system and primers csgB-LF and -LR were used as previously described to construct strain 43895Δ*csgB* (from here on referred to as Δ*csgB*) [37]. To generate the Δ*csgB*-complemented strain, PCR primers IBA-F and -R were used to amplify a 1704-bp fragment that included *csgBA* and its regulatory region. This fragment was digested with *Bam*HI/*Pst*I and cloned into pACYC177 to construct p*csgBA* that was transformed into strain 43895 Δ*csgB*. KAN^R^-recombinants were selected and tested for Congo red binding and biofilm formation.

### 2.2. Transposon Mutagenesis

The EZ-Tn*5* <KAN-2> Tnp Transposome kit (Epicentre Technologies, Madison, WI, USA) was used per the manufacturer’s instructions to generate biofilm mutants of strain 43895. KAN^R^-colonies were screened by a crystal violet biofilm assay (see below). Transposon insertion sites of biofilm-negative mutants were identified by PCR of flanking DNA using primers KAN-2 FP-1 or KAN-2 RP-1 (Table 2) followed by sequencing (MacrogenUSA, Rockville, MD, USA) and BLAST alignment.

### 2.3. Crystal Violet Biofilm Assay

The strain 43895 Tn*5* library was screened for biofilm-negative isolates. KAN^R^-colonies were inoculated into 96-well microtiter plates containing 200 µL LB-KAN/well. Wild type 43895 and biofilm-negative 43894 were positive and negative control strains, respectively. The plates were covered and incubated statically at 30 °C overnight. Liquid medium was removed, wells rinsed with PBS, attached bacteria were fixed (methanol/15 min), air dried, and stained with 200 µL of 1% crystal violet/5 min/room temperature. The plates were rinsed in water, air dried, and observed visually for crystal violet binding. O157 biofilm-negative mutants were confirmed by a tube pellicle-biofilm test, which shows crystal violet binding at the air-liquid interface. Briefly, mutants were grown at 37 °C, diluted 1:500 in 15-mL polystyrene tubes containing 4 mL LB, and incubated overnight at 37 °C without aeration. Liquid medium was removed, and the tubes were stained with 1% crystal violet for 5 min, rinsed with water, air dried, and compared visually to the controls.

Quantitative biofilm assays were performed as previously described [16]. Briefly, overnight cultures were diluted 1:50 into 96-well microtiter plates containing minimal salt medium with 1 mg/L yeast extract plus 0.4 g/L glucose and incubated statically at 30 °C or 37 °C for 24 h. Each strain was inoculated in triplicate. Following incubation, the plates were washed with water, stained with 1% crystal violet for 15 min at room temperature, and rinsed with water. The bound crystal violet was solubilized in 95% ethanol and quantified by absorbance at 595 nm using a PowerWave XS plate reader (Bio-Tek, Winooski, VT, USA).

### 2.4. Invasion Assay

A standard gentamicin protection assay [40,41] with minor modification was used to measure bacterial invasion of bovine epithelial cells. Briefly, 24-well tissue culture plates (Corning Costar, NY, USA) were seeded with 10^4^ bovine mammary epithelial cell line (MAC-T) cells and incubated in 5% CO_2_ at 37 °C until cells were confluent. MAC-T cell monolayers were washed twice with Hank’s Buffered Saline Solution (HBSS) and ~2 × 10^6^ O157 (MOI 10:1) in cell culture medium without antibiotics or fetal bovine serum (FBS; 1 mL) were added. After 3 h at 37 °C, unattached extracellular bacteria were removed by suction using a pipette and the monolayers were washed three times with HBSS. Fresh medium containing 100 µg/mL gentamicin (GEN) was added to kill extracellular O157. After an additional 2 h incubation, the monolayers were washed three times with HBSS without Ca^2+^ or Mg^2+^. Epithelial cells were lysed by adding 100 μL 0.5% trypsin-EDTA and 900 μL 0.05% Triton X-100/well for 5 min. Bacterial invasion was quantified by plate count as the CFUs recovered/well on LB agar as compared to 43894 and 43895 control strains.

### 2.5. Construction of csgD Promoter Mutants

Site-specific mutagenesis of 43894 and Sakai was used to change an A to a T in the *csgD* promoter (AATCT to ATTCT), creating 43894R and SakaiR, respectively. Lambda Red-mediated recombination was used for both strains 43894 and Sakai to replace 100 bp of DNA surrounding the *csgD* promoter that included the target sequence AATCT with a KAN^R^ cassette. The KAN^R^ cassette was amplified from pKD4 using primers Dele-1F and Dele-1R. The primers DBC1bF and DBC1bR were used to amplify DNA flanking the strain 43895 constitutive *csgD* promoter, and this fragment was then digested with *Pst*I/*Eco*RV to generate a 1012 bp fragment. This fragment was ligated into the pGP704 backbone (mob^+^, *sacBR*, AMP^R^, *pir*-dependent), digested with the same enzymes, and designated as pGP704csg1. Homologous recombination with pGP704csg1 was used to substitute the KAN^R^ cassette in both strains 43894 and Sakai with the 43895 *csgD* promoter (ATTCT) by conjugation using *E. coli* S17-1-*pir*-pGP704csg1 and selecting for plasmid co-integrates (AAM^R^ and KAN^R^). Resolution of pGP704csg1 co-integrates to generate the desired promoter mutation was accomplished by selecting for sucrose resistance on Congo red indicator plates containing 5% sucrose. Red colonies were selected and the bp change confirmed by amplifying the *csgD* promoter (primers csgDBign-F and csgDBign-R), DNA sequencing, and comparison to strains 43894 and Sakai (accession numbers AE005174 and BA000007).

### 2.6. Curli Isolation and Detection

O157 strains were grown in LB broth overnight at 37 °C without aeration. Bacteria were collected by centrifugation (5000× *g*/15 min/4 °C), and resuspended in PBS with protease inhibitor cocktail (Sigma-Aldrich), incubated on ice for 15 min. To shear fimbriae, cells were vortexed at 5.5 m/s for 20 s using a FastPrep FB120 Cell Disruptor (Abiogene, CA, USA). This vortexing was repeated 4 times with 10 min ice-incubations between each cycle. Cells were removed by centrifugation (8000× *g*/15 min/4 °C) and supernatants ultracentrifuged (41,000× *g*/3 h/4 °C) to sediment cell-surface fimbriae. To depolymerize the fimbriae, pellets were re-suspended in 99% formic acid (Sigma-Aldrich), incubated for 10 min on ice, vacuum dried, and re-suspended in Laemmli sample buffer (Bio-Rad Laboratories, Hercules, CA, USA). Proteins were separated by SDS-PAGE and transferred to PVDF membranes (Bio-Rad, Hercules, CA, USA) using a Mini Trans-Blot electrophoretic transfer cell (Bio-Rad). Curli subunits were detected by standard immunoblot using rabbit anti-CsgA sera (1:200; gift from M.R. Chapman, U of Michigan) and goat anti-rabbit-HRP conjugate (1:50,000; Sigma-Aldrich).

### 2.7. Operon Sequences

To investigate genetic variations responsible for biofilm/invasive-positive and -negative O157, we sequenced and compared the operons of *csgDEFG* and *csgBAC* of biofilm/invasive-negative strains 43894 and Sakai [16] to biofilm-positive strains 43895 and FB38. *E. coli* O157 FB38 is a bovine isolate that is *stx1* positive and *stx2* negative (Table 1).

### 2.8. Immunofluorescence Microscopy

MAC-T cells were grown overnight to ~80% confluence on 12 mm diameter coverslips in 24-well plates, individual O157 strains (2 × 10^6^ CFU) were added, mixtures incubated for 3 h, and washed five times with PBS. Cells were fixed with 4% paraformaldehyde/15 min, washed with PBS, permeabilized with 0.1% Triton X-100/4 min, and washed in PBS. The coverslips were incubated with rabbit anti-O157 (1:100; Denka Seiken CO., LTD, San Jose, CA, USA) or rabbit anti-CsgA (1:200) in PBS containing 2% bovine serum albumin, washed with PBS, and incubated for 1 h with Alex Fluor 488 chicken anti-rabbit antibody (1:100; Jackson ImmunoResearch, Inc. West Grove, PA, USA). To visualize the cytoskeleton, the permeabilized cells were incubated with rhodamine-phalloidin (Invitrogen, Carlsbad, CA, USA) for 40 min at room temperature. The cells on the slide were examined using fluorescence microscopy at 400× magnification.

### 2.9. Transmission Electron Microscopy

To examine epithelial cell invasion, MAC-T cells were grown overnight to ~80% confluence in 24-well tissue culture plates, individual O157 strains at 10 MOI were added and incubated for 3 h. MAC-T cells were washed with HBSS, removed from the plate using a cell scraper, and fixed in 2.5% glutaraldehyde/2% paraformaldehyde in 0.1 M cacodylate buffer, followed by 2% osmium tetroxide in 0.1 M cacodylate buffer for 1 h. The cells were dehydrated by passage through an ethanol series, rinsed with 100% acetone, infiltrated with Spurr’s resin, and polymerized overnight at 70 °C. Thin (90 nm) sections were cut and stained with 4% uranyl acetate and Reynolds lead.

To visualize fimbriae, 10 μL of overnight bacterial cultures were applied to 300-mesh carbon-formvar-coated copper grids for 2 min and negatively stained with 1% phosphotungstic acid (pH 7.4) for 5 min. To detect curli by immuno-electron microscopy, the grids were incubated with rabbit anti-CsgA antibody (diluted 1:50 in PBS, pH 7.4, containing 10% bovine serum albumin), rinsed, incubated for 1 h with goat anti-rabbit IgG conjugated to 10-nm gold particles, and washed as above. The grids were examined using A JEOL 1200 EX JEM transmission electron microscope at 10,000× magnification.

### 2.10. Cattle Challenge and O157 Enumeration

All biosafety and animal-handling protocols were approved by the University of Idaho Institutional Animal Care and Use and Biosafety Committees and strictly followed (approval number 2013-78; 6/7/2013–8/1/2016). Two groups of six, eight-month old Holstein steers were housed in a quarantined facility at the University of Idaho Agriculture Experiment Station, as previous described [42]. *E. coli* O157 naturally colonize at the bovine RAJ mucosa, so animals were both inoculated and sampled at this site. Animals in each group were given ~10^7^ CFU O157 (strains 43895 or 43895*csgB*::Tn*5*) in a single rectal application, as previously described [11]. Recto-anal mucosal swab (RAMS) samples were obtained from each steer on days 0 (prior to O157 challenge), 1, 3, 7, and twice a week thereafter, to 30 days post-challenge and processed for bacterial isolation, as previously described [11,43]. Briefly, RAMS samples were kept in 3 mL trypticase soy broth (TSB) on ice until processing. For direct cultures, serial 10-fold dilutions of the RAMS homogenates were plated on sorbitol MacConkey agar supplemented with cefixime (50 ng/mL), potassium tellurite (2.5 µg/mL), vancomycin (40 mg/liter), and 4-methylumbelliferyl-β-D-glucuronide (MUG, 0.1 mg/mL) (SMAC-CTVM). Plates were incubated overnight at 37 °C and O157 colonies (sorbitol-, MUG-) were counted. Latex agglutination confirmed the O157 antigen. RAMS samples, negative by direct culture, were enriched by overnight incubation prior to culture on SMAC-CTVM agar, and screened for O157, as above. RAMS samples negative on direct culture but positive by enrichment culture were arbitrarily assigned a value of 15 CFU/swab (equivalent to 50% of the minimum detectable direct-plating value). Strain persistence was determined as the percentage of cattle that remained continuously culture positive for O157. The proportion of animals that were culture positive for O157 from each group on each sampling day was compared.

### 2.11. Statistical Analysis

Graphs were drawn using Microsoft Excel and GraphPad Prism software 7 (San Diego, CA, USA). Counts of O157 recovered from gentamicin protection assays were transformed to log_10_ value, and their means compared using the *t* test procedure. The log-rank (Mantel–Cox) test was conducted to evaluate the statistical significance of the difference between durations of O157 carriage by cattle group.

## 3. Results

### 3.1. Tn5 Biofilm-Negative Insertions Map to LPS Synthesis or Curli Fimbriae Genes, but Only the Latter Class of Mutant Had Reduced Epithelial Cell Invasion

**Figure 1:**
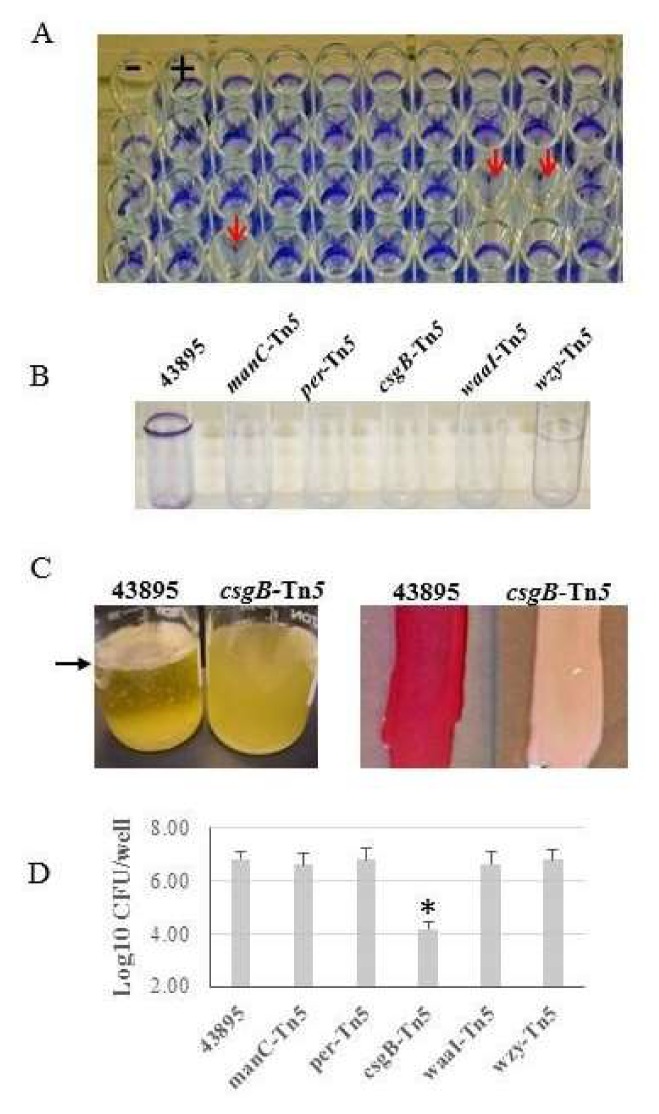
**O157 mutants lose biofilm, Congo red binding, and epithelial cell invasion ability**. (**A**) A Tn*5* insertion library of O157 strain 43895 (43895) was generated and screened using a crystal violet assay at 30 °C. A representative microtiter plate with Tn*5* mutants is shown with control strains 43894 (biofilm-negative, -) and 43895 (parental biofilm-positive, +); arrows indicate wells with biofilm-negative mutants. (**B**) Biofilm-negative mutants were confirmed by a static crystal violet tube assay at 37 °C as compared to the 43895 biofilm-positive control strain. Disrupted genes were identified by sequencing across the Tn*5*-insertion junction as *manC*, *per*, *csgB*, *waaI*, and *wzy*. (**C**) The *csgB*::Tn*5* mutant and the 43895 control strain were grown at 37 °C in Luria-Bertani (LB) broth without aeration to assess pellicle formation or on Congo red indicator agar to assess dye-binding. *csgB*::Tn*5* did not form a pellicle (left) at the air-liquid interface (arrow) and had reduced Congo red dye-binding (right). (**D**) Epithelial cell invasion was measured by a gentamicin protection assay. Bacteria were co-cultured with bovine mammary epithelial cell line (MAC-T) monolayers (MOI = 10) and CFUs determined by plate count (triplicate experiments, three replicates/strain). Only *csgB*::Tn*5* shows a ~100-fold reduction in cell invasion compared to 43895 and other biofilm-negative mutants; bars represent +SE and * denotes *p* < 0.05. The *csgB*::Tn*5* mutant had ~10-fold reduction in adherence to MAC-T cells compared to wild type 43895 (Table 3).

Strain 43895 produces a strong biofilm and invades epithelial cells, whereas O157 strains 43894, Sakai, and others do not [[16], and data not shown]. A Tn*5*-insertion library of strain 43895 was constructed and screened for loss of biofilm formation. Using a microtiter plate crystal violet assay, 43895 mutants were grown at 30 °C. Controls included strain 43894 and parental strain 43895. An initial screen of 1735 mutants were tested and five were found to have lost the ability to form biofilms. A representative assay plate is shown in Figure 1A. The genes responsible for conferring the biofilm negative phenotype were determined by DNA-sequencing across the Tn*5*-insertion junctions.

Two classes of biofilm-negative mutations were identified. The first class had insertions in genes required for lipopolysaccharide (LPS) biosynthesis including GDP-mannose mannosyl hydrolase (*manC*), perosamine synthetase (*per*), O-antigen polymerase (*wzy*), and lipopolysaccharide-ά-1,3-D galactosyltransferase (*waaI*; Figure 1B). These LPS mutants bound Congo red dye, but to a lesser degree than wild type strains 43895 (data not shown). The second class of mutation had a Tn*5*-insertion in *csgB*, required for curli formation (Figure 1B). The *csgB*::Tn*5* biofilm negative mutant lost the ability to bind Congo red dye compared with strain 43895 (Figure 1C).

To determine if biofilm formation was linked to epithelial cell invasion, each biofilm negative mutant was tested for the ability to invade bovine mammary epithelial cells (MAC-T) using a gentamicin protection assay. Strain 43895 and the mutants were individually co-cultured with MAC-T monolayers (MOI of 10:1) at 37 °C for 3 h. The number of bacteria recovered after 2 h gentamicin treatment was used to quantify invasion compared to strain 43895. Among all biofilm-negative mutants, only *csgB*::Tn*5* had a >100-fold reduction in cell invasion. The log-value of the intracellular *csgB*::Tn*5* mutant was 4.16; significantly lower (*p* < 0.01) than the 6.83 log-value of intracellular strain 43895 counts or the four LPS mutants (Figure 1D).

Based on these observations, clinical and ruminant isolates were screened for Congo red dye-binding, biofilm formation at 37 °C, and epithelial cell invasion. Among 12 clinical, 66 bovine, and 10 sheep isolates in our laboratory stock, one bovine isolate, FB38, and one outbreak strain were found to have these traits (data not shown). Moreover, pulse-field gel electrophoresis of *Xba*1-digest chromosomal DNA from strains 43894 and 43895 showed significant differences in the DNA digestion patterns (data not shown).

### 3.2. A Single Base Pair Change (A to T) in the csgD Promoter of O157 Strains Sakai and 43894, Conferred the Biofilm/Invasive-Positive Phenotype

**Figure 2:**
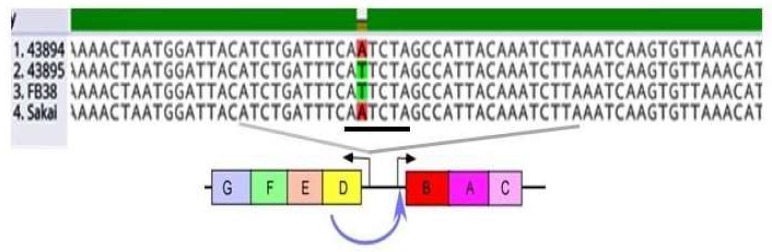
**Biofilm/invasive-positive and -negative O157 strains differ by a single nucleotide in the promoter of *csgD***. A schematic of the divergent curli operons and partial DNA sequences of the intergenic regulatory region of four O157 strains are shown. Biofilm/invasive-positive strains, 43895 and FB38, and -negative strains, 43894 and Sakai, differ by a single nucleotide A to T transversion (highlighted in green or red) in the *csgD* promoter. Below the sequences, a schematic depicting how regulation of curli subunits B and A are dependent on *csgD* expression (blue arrow).

To investigate genetic variations responsible for biofilm/invasive-positive and -negative O157, we sequenced and compared the operons of *csgDEFG* and *csgBAC* of biofilm/invasive-negative strains 43894 and Sakai [16] to biofilm-positive strains 43895 and FB38. *E. coli* O157 FB38 is a bovine isolate that is *stx1* positive and *stx2* negative (Table 1). We found the two biofilm/invasion-negative strains (Sakai and 43894) and the two biofilm/invasive-positive strains differed from the biofilm/invasion positive strains (FB38 and 43895) by a single nucleotide A to T transversion of the putative *csgD* promoter (Figure 2).

**Figure 3:**
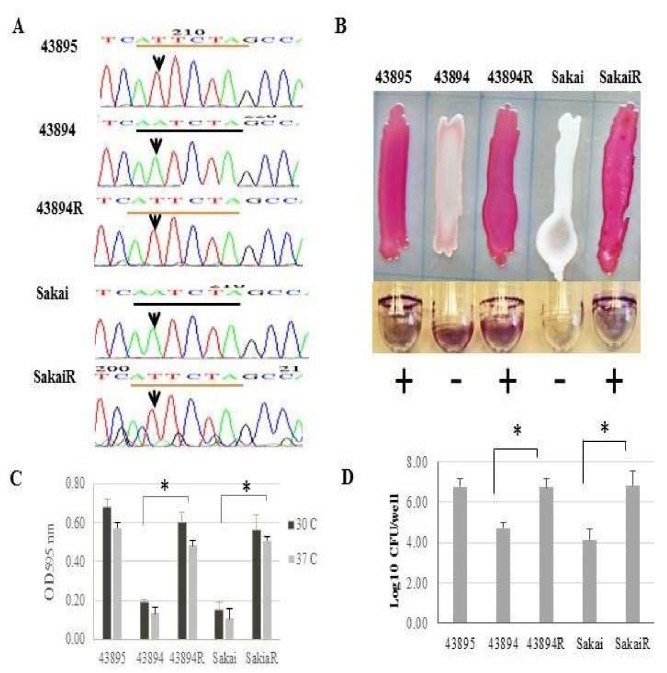
**A single nucleotide change in the O157 *csgD* promoter confers Congo red binding, biofilm production, and invasion phenotypes**. Site-directed mutagenesis was used to generate an A to T transversion in the curli regulatory region of 43894 and Sakai, designated 43894R and SakaiR, respectively. (**A**) The DNA sequence of parental and mutagenized strains are compared to 43895. The arrow denotes the T or A of each strain. (**B**) Congo red dye-binding and biofilm formation were compared by growing each strain on Congo red indicator agar or in LB broth without aeration at 37 °C, respectively. The single base pair changes in 43894R and SakaiR conferred dye-binding and biofilm formation (+ or -) equivalent to 43895. (**C**) Biofilm formation was quantified by a microtiter assay. Strains were incubated at 30 °C or 37 °C in minimal salts media with 4% glucose. Bound crystal violet was solubilized with 95% ethanol and measured by absorbance at 595 nm. Assays were performed six times/strain. Bars represent +SE and * denotes *p* < 0.05. At both temperatures, the single base change in 43894R and SakaiR conferred biofilm formation equivalent to 43895. (**D**) Epithelial cell invasion was measured by a gentamicin protection assay. Bacteria were co-cultured with MAC-T monolayers (MOI = 10) and CFUs determined by plate count (triplicate experiments, three replicates/strain). The single base change in 43894R and SakaiR conferred epithelial cell invasion similar to 43895. Bars represent +SE and * denotes *p* < 0.05.

To determine the significance of the single base variation in *csgD* promoter, site-directed mutagenesis was used to change A to T in the *csgD* promoter of both strains 43894 and Sakai to match strain 43895. The mutated strains were designated 43894R and SakaiR, respectively. The point mutations were confirmed by DNA sequencing (Figure 3A). The single base pair change in strains 43894 and Sakai conferred Congo red dye-binding and biofilm formation at 37 °C, similar to strain 43895 (Figure 3B,C). Strains 43894R and SakaiR invaded epithelial cells as determined by a MAC-T gentamicin protection assay. A 100-fold increase in intracellular 43894R and SakaiR compared to the parental wild type strains 43894 and Sakai was measured (Figure 3D). Thus, the single base A to T transversion resulted in the pleiotropic phenotypic change to biofilm/invasive-positive and Congo red dye-binding.

### 3.3. Curli Fimbriae Are Expressed in O157 Strains 43895, 43894R, and SakaiR but Not in Wild Type Strains 43894, Sakai, or the Mutant csgB::Tn5

**Figure 4:**
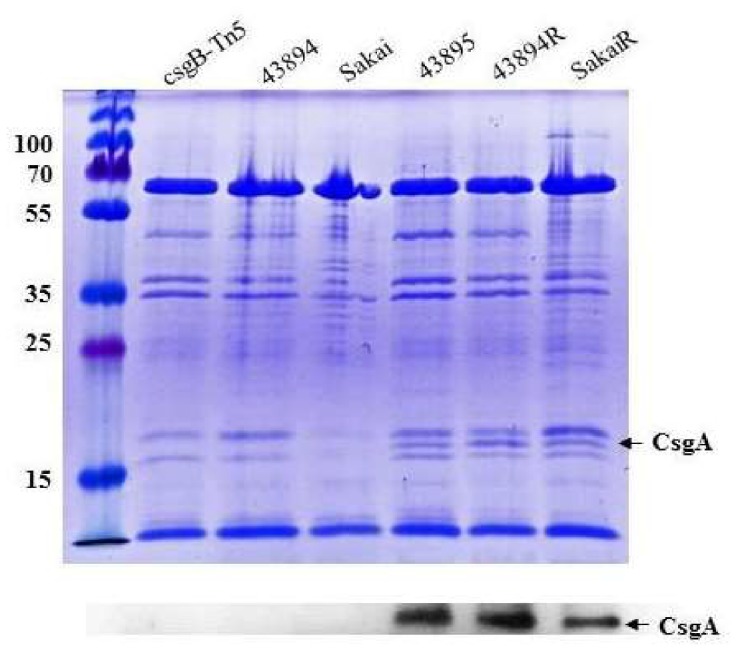
**Curli fimbriae were expressed in O157 invasive strains but not in non-invasive strains**. Bacterial strains were grown overnight in LB broth at 37 °C without aeration. To detect the major subunit of curli fimbriae CsgA, cell surface fibers were sheared by vortexing, concentrated by ultra-centrifugation, depolymerized with formic acid, and separated by SDS-PAGE. Proteins were visualized by Coomassie blue straining or transferred to membranes for immunoblotting. The predicted CsgA 17-kDa protein is seen only in 43895, 43894R, and SakaiR (CsgA arrow; molecular weight standards and kDa values are at left). The CsgA identification is confirmed by immunoblot with anti-CsgA sera (CsgA arrow).

Congo red dye-binding by *E. coli* correlates with the amyloid protein curli fimbriae on the cell surface [44]. Because the single base-pair change in the *csgD* promoter conferred Congo red dye-binding in both strains 43894R and SakaiR, we confirmed the presence of curli on the bacterial surface by immunoblot. Briefly, bacteria were grown without aeration at 37 °C, cell surface fibers were sheared by vortexing, concentrated by ultra-centrifugation, depolymerized with formic acid, and separated by SDS-PAGE. Proteins corresponding to the size of CsgA monomers were present on the surfaces of strains 43895, 43894R and SakaiR, but not in strains 43894, Sakai, or the mutant *csgB*::Tn*5*, and were confirmed by immunoblot with anti-CsgA antibody (Figure 4). CsgA, an amyloid protein, migrates at a higher molecular weight than its predicted size. Anti-CsgA sera binds strongly to this protein band.

### 3.4. Deletion of csgB Resulted in the Same Phenotype as the csgB::Tn5

**Figure 5:**
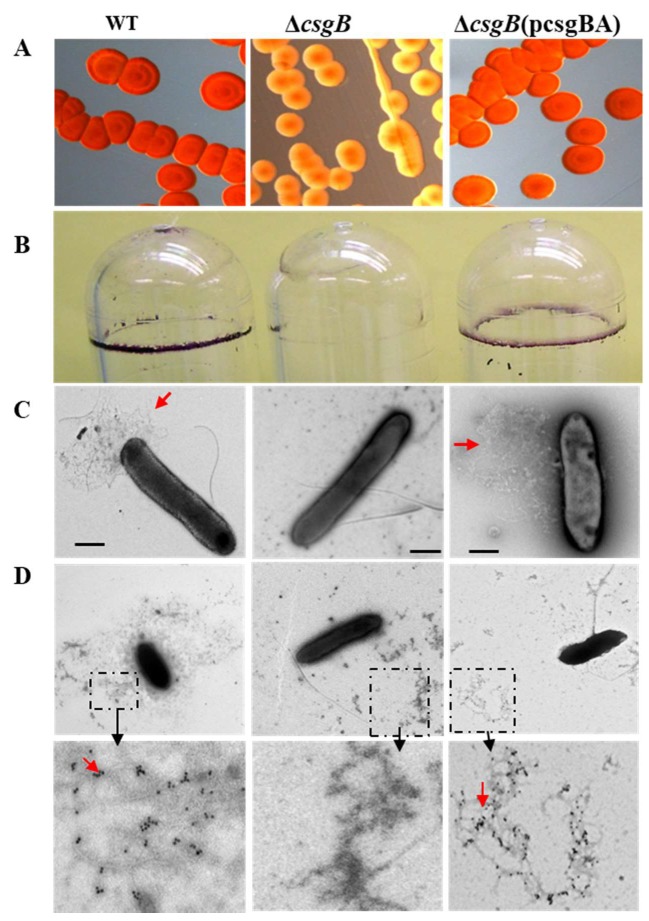
**Δ*csgB* does not bind Congo red dye, does not form biofilm, and lacks curli fimbriae**. (**A**) Bacterial overnight cultures of 43895 (WT), Δ*csgB*, and the complement Δ*csgB*(pcsgBA) were plated on Congo red indicator agar, incubated at 37 °C for 24 h, and examined visually. Colonies of Δ*csgB* were less red than the colonies of the WT and Δ*csgB*(pcsgBA) controls. (**B**) Bacterial cultures of the same strains were grown overnight, statically in LB at 37 °C. Medium was removed and pellicle-biofilms detected by staining with 1% crystal violet. The Δ*csgB* culture did not form a pellicle-biofilm as did the cultures of the WT and Δ*csgB*(pcsgBA) controls. Static overnight cultures of the same strains were individually applied on 300-mesh carbon-Formvar-coated copper grids and prior to EM examination (original magnification 10,000×) were (**C**) negatively stained with 1% phosphotungstic acid, pH 7.4 or (**D**) immunostained with rabbit anti-CsgA sera and goat anti-rabbit IgG conjugated with10-nm gold particles. Arrows point to anti-CsgA immunogold deposited onto curli fibers in the enlarged dash boxes. No curli fimbriae were detected with Δ*csgB*.

To confirm that the *csgB*::Tn*5* mutant characteristics were not due to multiple transposon insertions or polar insertional effects, an in-frame deletion of *csgB* was made in strain 43895 using site-directed mutagenesis and designated Δ*csgB*. Moreover, the *csgB* gene was cloned on a plasmid, designated p*csg*BA. The Δ*csgB* mutant lost Congo red dye-binding compared to the wild type strain 43895 and the complemented strain Δ*csgB*(p*csgBA*) (Figure 5A). The Δ*csgB* mutant strain did not form a pellicle-biofilm but the complemented and parental strains did (Figure 5B). Thus, the independent, single *csgB* mutation resulted in the same pleotropic phenotypic losses of the *csgB*::Tn*5* mutation.

Curli fimbriae were visualized on the bacterial surface using immuno-electron microscopy (EM) with gold-labeled anti-CsgA. Curli was observed on the cell surface of strains 43895 and Δ*csgB*(p*csgBA*) but not the Δ*csgB* mutant cells (Figure 5C). Curli, other fimbriae, and flagella are easily sheared during EM sample processing, but the material is fixed to the EM grids. Only fibrous material surrounding the strains 43895 and Δ*csgB*(p*csgBA)* reacted with antiCsgA immuno-gold particles. In contrast, the fimbriae surrounding the mutant Δ*csgB* did not react with anti-CsgA immune-gold particles (Figure 5D).

### 3.5. Curli Was Required for O157 Adherence to and Invasion of MAC-T Cells

**Figure 6:**
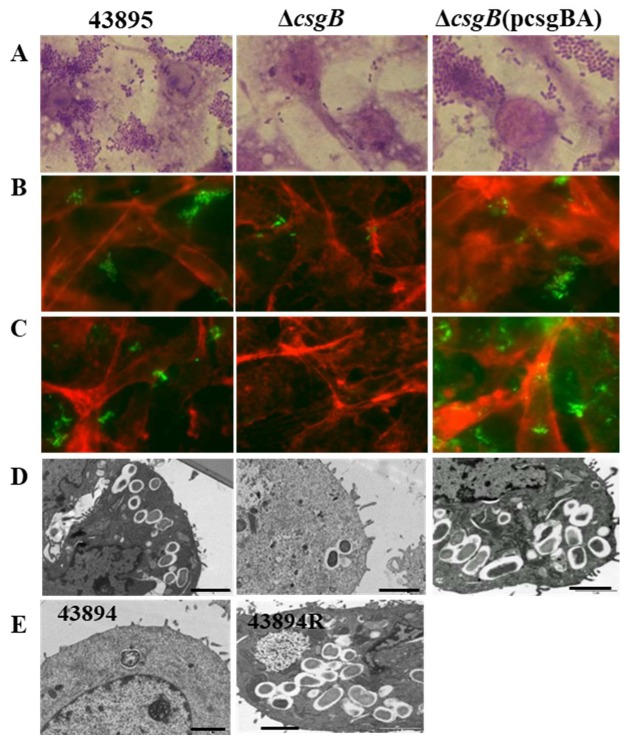
**The role of curli in adherence to and invading MAC-T cells**. Individual overnight cultures of *E. coli* O157:H7 43895, curli-negative Δ*csgB*, the complemented strain Δ*csgB*(pAB), and 43894R bacterial strains were co-cultured with MAC-T monolayers on coverslips at MOI = 10 and incubated for 3 hrs. Microscopy images show interaction of *E. coli* O157:H7 43895 and curli-negative mutant Δ*csgB*, the complemented strain Δ*csgB*(pAB), 43894 and 43894R with MAC-T monolayers. Curli-negative Δ*csgB,* showed a ~10-fold reduction in adherence compared to wild type 43895 (data not shown and Table 3) (**A**) Giemsa stain showing the 43895 aggregative adherence pattern to MAC-T monolayers compared to binding of a Δ*csgB* mutant (inverted microscope, magnification × 1000). (**B**,**C**) Immunofluorescence showing bacteria interact with MAC-T cells (magnification × 1000) stained by (**B**) anti-O157 LPS antibody (green) or (**C**) anti-curli antibody (green), the host cell cytoskeleton was stained with phalloidin-TRITC (red). (**D**,**E**) TEM showing extracellular and intracellular bacteria (strains indicated at top left of each micrograph) adhered to or within the vacuoles of MAC-T cells (magnification, ×10,000). Ruler bars in D and E = 2 µm.

We previously showed O157 43895 has a strong ability to adhere to and invade bovine epithelial cells compared to 43894 and other *E. coli* O157 strains [16]. Here, the role of curli in epithelial cell invasion was examined. Strain 43895, its isogenic mutant Δ*csgB*, and the complemented strain Δ*csgB*(p*csgBA*) were individually co-cultured with MAC-T cells. The Δ*csgB* mutant showed reduced adherence to MAC-T cells compared to the parental and complemented strains (Figure 6A). Similar bacterial adhesion patterns were visualized by immunofluorescence using anti-O157 LPS (Figure 6B) or anti-curli (Figure 6C).

Transmission EM of the co-cultured MAC-T cells had intracellular bacteria within membrane-bound vacuoles of the parental wild type strain 43895 and the Δ*csgB*(p*csgBA*) strain (Figure 6D,E). The number of intracellular strain 43895 or Δ*csg* -complemented bacteria ranged from 10 to 15 per cell, while the Δ*csgB* mutant was rarely internalized by MAC-T cells. These observations were supported by bacterial plate counts (Table 3). The Δ*csgB* mutants had a ~10 fold reduction in adherence to MAC-T cells compared to wild type strain 43895 or Δ*csgB*(p*csgBA*) strains (Table 3). In gentamicin protection assays, the number of recovered bacteria of Δ*csgB* mutants was reduced by more than 100-fold compared to wild type or the Δ*csgB*(pcsgBA) complemented strain (*p* < 0.01).

### 3.6. The csgB::Tn5 Mutation Reduced Persistence of E. coli O157 Strain 43895 in Cattle

**Figure 7:**
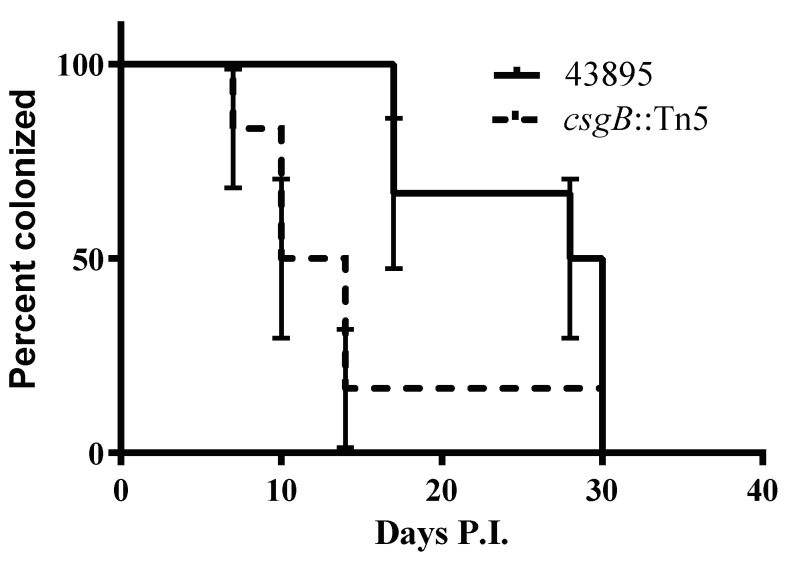
**Curli production enhances persistence of O157 in cattle**. Two groups of six cattle were challenged with WT 43895 and the isogenic curli-negative mutant *csgB*-Tn5 using a single rectal application of 10 CFU of bacteria. Rectal swab samples from each animal was taken twice a week and cultured for *E. coli* O157 for 30 days. Percent survival indicated the percent of the cattle that were *E. coli* O157 culture positive. Survival time (duration of carriage) was defined as the number of days animals were cultured positive with *E. coli* O157 and the *p* < 0.05 was calculated by the log-rank (Mantel–Cox) test.

Persistence in cattle by the curli-negative *csgB*::Tn*5* mutant and the isogenic curli-positive wild type strain 43895 was compared. Two groups of six eight-month-old Holstein steers received a single rectal dose of 10^7^ CFU of 43895 or the isogenic *csgB*-Tn*5* derived mutant. Colonization at the terminal rectal mucosa was monitored by culture of *E. coli* O157 in RAJ mucosal swab (RAMS) samples from each steer twice a week for 30 days post-challenge. The duration of bacterial persistence among the strain 43895-challenged animals compared to *csgB*::Tn*5* mutant-challenged animals was different as determined using the log-rank (Mantel–Cox) test (*p* < 0.05). The median duration of carriage of strain 43895 was 29 days, whereas the median duration of carriage of *csgB*::Tn*5 isogenic* mutant was 12 days (Figure 7). This finding indicated that curli enhanced persistence of O157 in cattle.

## 4. Discussion

This work independently confirmed and extended findings about the role of curli fimbriae in diverse and important phenotypes of O157 [45]. Transposon mutagenesis revealed two classes of biofilm-negative mutants, one dependent on LPS and the other dependent on curli. A Tn*5* insertion in the *csgB* structural curli gene resulted in a pleiotropic phenotype affecting O157 biofilm formation, epithelial cell invasion, Congo red dye-binding, and persistence in cattle. In contrast to Uhlich et al., curli expression in strain 43895 (curli-positive) and strains 43894 (curli-negative) was stable and there was no phenotypic variation in these strains. Site-directed mutagenesis (A to T) of the *csgD* curli promoter converted two curli-negative strains (43894 and Sakai) to curli-positive with stable expression of the full complement of curli-associated phenotypes.

Uhlich et al. report that curli expression in O157 strains 43894 and 43895 is variable and changes at a frequency of 10^−4^ [34]. This ‘phase variation’ is identified as a mixture of distinct red and white colonies after subculture by plating on Congo red indicator agar. The event is ascribed to an A to T transversion in the *csgD* promotor. However, a phenotypic change at this frequency should result in sectored or papillated red-white colonies. Phenotypic changes in bacteria at a 10^−4^ frequency occur through the mechanisms of transposition, DNA rearrangement, loss of a plasmid, or acquisition of a mutator phenotype like *mutD* or *mutS*. A single base transversion, like the A to T change described here, occurs at a much lower frequency of ~10^−9^ and would be stable. Using stock strains and freshly acquired strains from ATCC, we found that this change was stable, and strains did not display phenotypic (phase) variation. To verify this stability and to definitively show the pleiotropic effect of the single base pair difference in strains 43894 and 43895, we used site-directed mutagenesis. Reproducing the A to T transition in the *csgD* promoter in both strains 43894 and Sakai clearly show the pleiotropic phenotype and the stability of each converted strain. Moreover, pulse-field gel electrophoresis band patterns of strains 43894 and 43895 indicated they are two different strains. Thus, a population of O157 with variable curli expression most likely includes more than one O157 strain.

Assessing curli expression may be important for determining the pathogenic potential of a strain and this work showed that neither Congo red dye-binding nor detection of fimbriae by EM are definitive assays. Insertions in LPS O-antigen or outer-core biosynthetic genes reduced Congo red dye-binding, even when curli fimbriae were still expressed. Similar curli expression occurs in *E. coli* K12 mutants [18]. The *csgB*::Tn*5* mutant failed to express curli but other pili-like material was visible by EM. Reliable assessment of curli expression was accomplished with a rapid method to extract all large polymeric fibers (fimbriae and flagellin) and immunoblotting with curli-specific antibody.

To our knowledge, this is the first study to evaluate the role of curli expression in persistence of O157 in cattle in a controlled study. The most powerful approach to determine if a gene and its protein product contribute to persistence is to compare the effect of a specific mutation to its isogenic parent. That is what was done here. When strain 43895 was compared to its isogenic curli-negative derivative, it persisted significantly longer at the RAJ site of cattle. We concluded that the curli fimbriae enhanced persistence of O157 in cattle. This conclusion is corroborated by our previous observation that strain 43895 persists in cattle longer than the non-isogenic curli-negative strain 43894 [16]. Possible mechanisms for the role of curli in animal carriage include curli promotion of adherence to epithelial cells and curli-mediated crypt cell internalization [16]. Increased persistence in cattle could impact environmental, hide, and carcass contamination, with resulting higher virulence in human clinical infections.

In an estimated 95% of O157, curli synthesis is highly attenuated or absent because the Shiga toxin type 1-prophage inserts in *mrlA*, a positive activator that is required for *csgD* expression [45]. *mrlA* is under σ^S^ control so that among non-O157 *E. coli*, curli is normally expressed under stationary phase conditions. In the case of O157 with the single base pair A to T transversion in the promotor of *csgD*, curli expression is converted from σ^S^-dependence to the normal house-keeping σ^D^ dependence [45] resulting in constitutive curli expression in certain O157 strains. Thus, the association between O157 virulence and absence of *stx*-1 [46] may be partly explained by lack of curli expression.

These and others’ findings link the single base pair (A to T) transversion in the *csgD* promoter to a pleiotropic phenotype. The mechanism for this change may be due to altered local DNA folding. Computer modeling using Geneious R11 (https://www.geneious.com) shows significantly different tertiary structure in the intergenic sequence between the *csgD* and *csgA* operons. Whether or not these computer models accurately predict changes in structure that would favor *rpoS* or *rpoD*, the biological consequence is a change from stationary phase expression only to constitutive expression of *csgD*. A similar change in the *Salmonella* Typhimurium *csgD* promoter converted tightly regulated curli expression to constitutive expression similar to these results with O157 [47].

Thus, the use of site-specific mutagenesis definitively showed that multiple O157 phenotypes contributing to persistence in cattle and the environment and impacting virulence come from a single base pair change in the *csgD* promoter.

## 5. Conclusions

O157 is an emerging pathogen. At least one step in its trajectory from a commensal to causing human disease was the acquisition of lysogenic phage carrying a Shiga toxin (Stx) gene. Insertion of the Stx-1 prophage in the chromosome disrupts expression of curli fimbriae. These pili are known to promote biofilm formation and bacterial invasion into eukaryotic cells. With few exceptions, O157 does not produce biofilm at 37 °C, nor invade cells. Both lost attributes, if re-gained, may increase virulence or persistence in the environment. The rare curli-producing O157 strains show strong biofilm formation at 37 °C and the ability to invade epithelial cells. This correlates with a single base pair transversion (A to T) in the curli promoter. The importance of our work is that we showed by site-specific mutagenesis that this single base pair change converts strains from curli-negative to constitutive curli expression at 37 °C. This mutation is stable and also confers bacterial persistence in healthy cattle, the natural reservoir for this human pathogen. Reemergence of curli constitutive expression could increase virulence by introducing the pleiotropic phenotype of biofilm formation, host cell invasion, and persistence in cattle. Epidemiological screening for curli could be important.

## Figures and Tables

**Table 1:** *E. coli* strains and plasmids.

Strain or Plasmid	Description	Source or Reference
43895	*E. coli* O157:H7 ATCC 43895, a clinical isolate, *stx1*+/*stx2*+, curli+	ATCC *
*manC*-Tn*5*	Tn*5* inserted in *manC* of 43895	This work
*per*-Tn*5*	Tn*5* inserted in *per* of 43895	This work
*csgB*-Tn*5*	Tn*5* inserted in *csgB* of 43895	This work
*waaI*-Tn*5*	Tn*5* inserted into *waaI* of 43895	This work
*wzy*-Tn*5*	Tn*5* inserted into *wzy* of 43895	This work
43895Δ*csgB*	43895 with *csgB* deletion	This work
43895Δ*csgB*(pcsgBA)	43895Δ*csgB* complemented *csgB*	This work
43894	*E. coli* O157:H7 ATCC 43894, a clinical isolate, *stx1*+/*stx2*+, curli-	ATCC
43894R	43894 mutant with A to T transversion in *csgD* promoter, curli+	This work
Sakai SakaiR	*E. coli* O157:H7, a clinical isolate, *stx1*+/*stx2*+, curli- Sakai mutant with A to T transversion in *csgD* promoter, curli+	[16] This work
FB38	*E. coli* O157:H7, a bovine isolate, *stx1*+/*stx2-,* curli positive	Laboratory stock
S17-1 λ pir	*pro recA thi hsdR* Hfr RP4-2 (Tc::Mu) (Km::Tn7, SmR, λ pir lysogen	[38]
K-12	*E. coli* MG1655 strain	Laboratory stock
pKD4	Template plasmid for mutagenesis (AmpR KanR)	[37]
pKD3	Template plasmid for mutagenesis (AmpR CmR)	[37]
pKD46	Red recombinase helper plasmid, RepA101(Ts), AmpR	[37]
pACYC177	Cloning vector, AmpR, KanR	ATCC
pGP704.L	Suicide plasmid, mob^+^, sacBR^+^, pir-dependent oriR6K, AmpR	[39]
pGP704csg1	pGP704.L containing *csgBA* genes of 43895; allelic exchange plasmid	This work
pcsgBA	*csgBA* and intergenic region of 43895 cloned into pACYC177	This work

* ATCC, American Type Culture Collection, Manassas, VA.

**Table 2:** Oligonucleotide primers.

Primer	* Sequence (5′–3′)
KAN-2 FP-1	ACCTACAACAAAGCTCTCATCAACC
KAN-2 RP-1	GCAATGTAACATCAGAGATTTTGAG
IBA-F	GTTTGGATCCAAACCCCGCTTTTTTTATTGATC (*Bam*HI)
IBA-R	GTTTCTGCAGTTAGTACTGATGAGCGGTCGCGT (*Pst*I*)*
csgDBign-F	GAGCCTGAAGAGATATCGTCCA
csgDBign-R	GCGCACCCAGTATTGTTAAC
csgB-LF	ATGAAAAACAAATTGTTATTTATGATGTTAACAATACTGGGTGTGTAGGCTGGAGCTGCTTCG
csgB-LR	TTAACGTTGTGTCACGCGAATAGCCATTTGCGACTGTCTCTGCATATGAATATCCTCCTTA
Dele-1F	AGAAGTACTGACAGATGTTGCACTGCTGTGTGTAGTAATAAATGTGTAGGCTGGAGCTGCTTCG
Dele-1R	AACTTAATAAAACCTTAAGGTTAACATTTTAATATAACCAGTCATATGAATATCCTCCTTA
DBC1bF	TATACCCGGGTTCTTGATCCTCCATGGCATAAAA (*Sma*I)
DBC1bR	ATATAGATATCCTGCGTTACGATGGAAAGTATGTC(*Eco*RV)

* Restriction enzyme sites are underlined.

**Table 3:** Adherence to and invasion of MAC-T cells by O157 strain 43895 and its derivatives.

Strains ^a^	Cell-Associated ^b^ (CFU × 10^7^/Well)	Internalized ^c^ (CFU × 10^4^/Well)
43895	8.78 ± 0.73	467 ± 190
Δ*csgB*	* 0.71 ± 0.12	** 4.58 ± 0.89
Δ*csgB*(p*csgBA*)	7.66 ± 0.67	389 ± 110

^a^ Experiments were done in triplicate. ^b^ CFU recovered from washed, lysed monolayers after 3 h incubation with bacteria. ^c^ CFU recovered from lysed monolayers after 3 h incubation with bacteria and 2 h gentamicin treatment. * *p* > 0.05, ** *p* < 0.01 determined by Student’s t test.
